# High-Performance Self-Powered Dual-Mode Ultraviolet Photodetector Based on (PEA)_2_PbI_4_/GaN Heterojunction

**DOI:** 10.3390/nano14221819

**Published:** 2024-11-13

**Authors:** Ang Bian, Songchao Shen, Chen Yang, Jun Dai

**Affiliations:** School of Science, Jiangsu University of Science and Technology, Zhenjiang 212100, China; shensongchao@stu.just.edu.cn (S.S.); yangchen@stu.just.edu.cn (C.Y.)

**Keywords:** perovskite, wide-bandgap semiconductor, ultraviolet detector, heterojunction

## Abstract

Wide-bandgap semiconductors like GaN, known for their superior photoresponse and detection capabilities in the ultraviolet range, represent a foundational component in the design of advanced photodetectors, where the integration of materials with distinct spectral sensitivities into heterojunctions is pivotal for next-generation device innovation. A high-performance self-powered dual-mode ultraviolet photodetector based on a (PEA)_2_PbI_4_/GaN heterojunction was fabricated via spin coating. The device exhibits outstanding UV sensitivity under both positive and negative bias, achieving a responsivity of 1.39 A/W and a detectivity of 8.71 × 10^10^ Jones under 365 nm UV illumination. The built-in electric field at the heterojunction interface enables self-powered operation, achieving a rapid rise time of 46.9 ms and a decay time of 55.9 ms. These findings offer valuable insights into the development and application of perovskite and wide-bandgap semiconductor heterojunctions in optoelectronic devices.

## 1. Introduction

Ultraviolet (UV) photodetectors are critical components for the future of intelligent optoelectronic systems, with broad applications across various fields, including optical communication, military detection, industrial inspection, security monitoring, healthcare, and environmental monitoring [1]. As one of the most important UV optoelectronic devices, the demand for high-performance UV photodetectors has been rapidly increasing, making them a focal point in the field of photodetector research, which has seen significant advancements in recent years [2,3,4]. Wide-bandgap semiconductors, characterized by a bandgap width greater than 2.3 eV, such as GaN [5], SiC [6], diamond [7], and Ga_2_O_3_ [8], exhibit high sensitivity to UV light. These stable and durable advanced materials can effectively absorb UV radiation and generate electron–hole pairs for efficient photoelectric conversion, making them ideal UV detection materials [9,10,11]. However, despite the excellent performance of single materials in UV detectors, there are still certain aspects that require improvement.

Perovskite materials represent a novel class of optoelectronic materials that have garnered significant attention in recent years due to their outstanding properties, including high photoelectric conversion efficiency, wide absorption spectrum, and superior carrier mobility [12]. Structurally, perovskites are defined by their ABX_3_ crystal structure and are known for their low cost, optimal bandgap width, and status as direct bandgap materials [13]. They exhibit an extremely high extinction coefficient, long carrier diffusion length, and excellent light absorption capabilities, surpassing many organic optoelectronic materials [14]. In recent years, the stability of two-dimensional layered organic perovskites has improved significantly, particularly under X-ray radiation, where they exhibit exceptional stability [15]. Additionally, perovskites offer excellent bipolar carrier transport characteristics, making them promising candidates for high-performance photodetectors [16].

Currently, heterojunction photodetectors integrating wide-bandgap semiconductors with other semiconductor materials have demonstrated remarkable performance and distinct advantages, yet this area of research is still in its infancy. For instance, Zhou H et al. fabricated the first photodetectors based on a CH_3_NH_3_PbI_3_/GaN heterojunction, exhibiting high stability and self-powered characteristics [17]. Zdanowicz et al. conducted a preliminary study on the Fermi level contact of the MAPbI_3_/GaN heterojunction [18]. Zhou X et al. achieved a high-performance self-powered ultraviolet photodetector by growing nontoxic copper halide perovskite CsCu_2_I_3_ thin films on a GaN substrate using the vacuum thermal evaporation method [19]. Furthermore, ultraviolet photodetectors based on perovskite and other traditional semiconductor heterojunctions also exhibit good performance, such as Ga_2_O_3_ [20] and GaAs [21]. Nevertheless, these wide-bandgap semiconductors have limitations in terms of light absorption and carrier transport efficiency. Therefore, it remains crucial to explore the performance enhancement pathways of heterojunction photodetectors by selecting easily processed perovskite materials with high absorption coefficients.

In this study, we developed a heterojunction UV photodetector based on Ruddlesden-Popper (RP) perovskite and GaN. Using spin-coating methods, we constructed a novel (PEA)_2_PbI_4_/GaN heterojunction diode, with the p-type (PEA)_2_PbI_4_ vertically layered on an n-type GaN substrate. This heterojunction exhibits type-II band alignment. UV photoelectric tests revealed that under both positive and negative voltages, the detector exhibited a strong response to 365 nm UV light, enabling dual-mode operation. The measured responsivity (*R*) under positive voltages were both highly favorable. Thanks to the built-in electric field at the heterojunction interface, the detector also operates effectively in self-powered mode with rapid response times, which is significant for energy efficiency and remote applications. This study highlights the significant development potential of photodetectors based on (PEA)_2_PbI_4_/GaN heterojunction diodes, offering innovative pathways for the advancement of intelligent optoelectronic systems.

## 2. Materials and Methods

Prior to device fabrication, the n-GaN crystal substrate was successively cleaned in an ultrasonic bath with deionized water, acetone, isopropanol, and anhydrous ethanol for 5 min. (PEA)_2_PbI_4_ films were deposited by a one-step spin-coating method. The thickness control is primarily influenced by several factors, including the wettability of the substrate, spin-coating speed, precursor volume, and precursor concentration. In short, (PEA)_2_PbI_4_ films were prepared by antisolvent extraction in a N_2_ glove box. The (PEA)_2_PbI_4_ precursor solution was prepared by dissolving 124.5 mg of PEAI and 115.3 mg of PbI_2_ in 1000 μL DMF(N,N-dimethylformamide) solution. Afterward, 80 μL of the precursor solution was added onto the n-GaN single crystal substrate, which is around 1 × 1 cm^2^ framed by the tape, at a rotation speed of 2000 rpm for 30 s. Then, it was rapidly thermally annealed at 100 °C for 15 min in the same environment.

Based on the obtained (PEA)_2_PbI_4_/GaN planar heterojunction, cylindrical indium (In) metal pieces were mechanically transferred onto the surfaces of both the GaN and (PEA)_2_PbI_4_ thin films to serve as electrodes [22]. Since GaN is an n-type semiconductor and (PEA)_2_PbI_4_ is typically a p-type semiconductor, the resulting structure is a p-n junction photodiode for UV detection.

The absorption spectrum and optical bandgap of the heterojunction sample were measured using a HITACHI U-4100 ultraviolet-visible (UV-Vis) spectrophotometer (HITACHI, Tokyo, Japan). The X-ray diffraction (XRD) patterns of the sample powders were recorded using a Rigaku Corporation Smart Lab diffractometer (Rigaku, Akishima, Japan). The surface morphology and cross-section of the samples were examined using a scanning electron microscope (SEM) of FEI Quanta FEG 250 (Tokyo, Japan). To investigate the UV photodetection properties, current–voltage (I–V) measurements were conducted in air at room temperature using a Keithley 4200-SCS semiconductor parameter analyzer system (Tektronix, Beaverton, OR, USA). Photocurrent measurements dependent on voltage and UV light intensity, as well as time-dependent response tests, were performed using an intensity adjustable UV light-emitting diode (LED) at a wavelength of 365 nm. The light emitted from the source is transmitted through a light-shielding tube and focused by the objective lens to vertically illuminate the heterojunction region between the two electrodes on the sample. The resulting spot area corresponds to the effective illuminated area, which is approximately 0.2 cm^2^.

## 3. Results and Discussion

**Figure 1:**
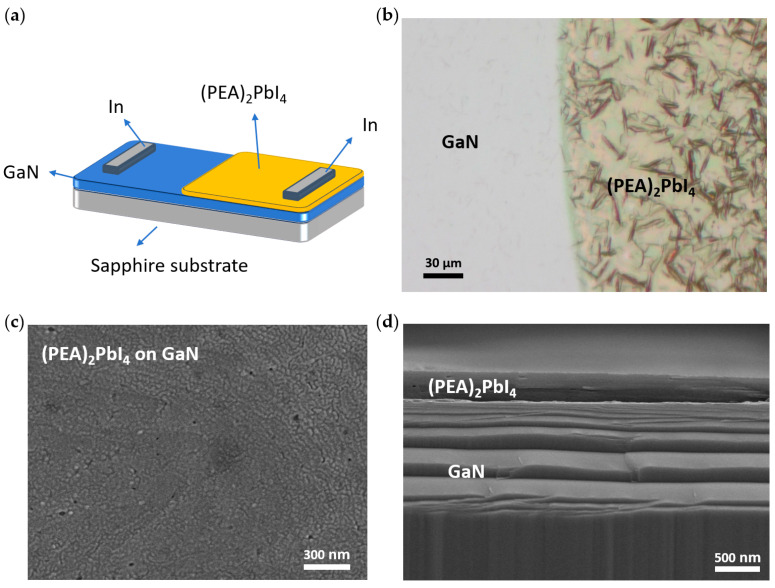
(PEA)_2_PbI_4_/GaN heterojunction photodetector. (**a**) planar structure, (**b**) optical image, (**c**) the top-view SEM image, (**d**) cross-section SEM image.

The schematic diagram of the (PEA)_2_PbI_4_/GaN heterojunction photodetector structure is shown in Figure 1.

Figure 1b shows the (PEA)_2_PbI_4_ film spin-coated on the GaN wafer after masking, illustrating the clean GaN regions and the heterojunction areas covered with (PEA)_2_PbI_4_. The top-view and cross-section SEM images are shown in Figure 1c,d, respectively. The prepared (PEA)_2_PbI_4_ exhibits a smooth surface and uniform interface, with a thickness of approximately 300 nm.

**Figure 2:**
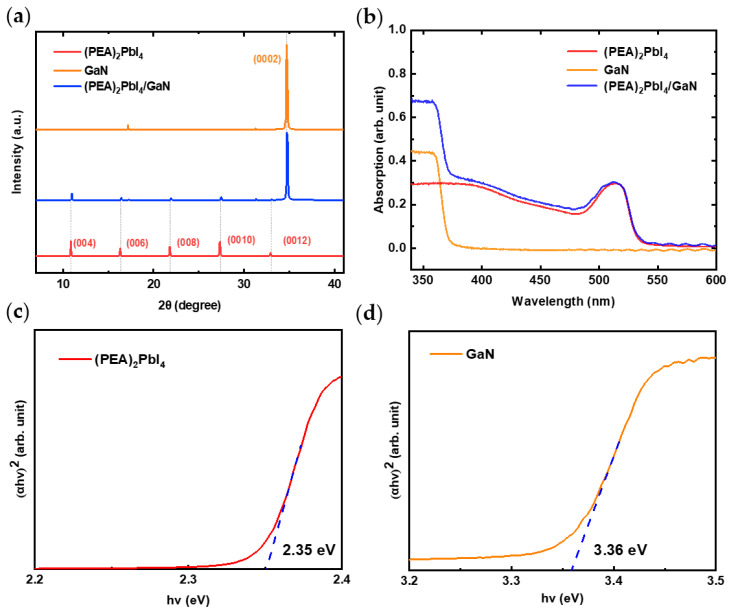
(**a**) XRD pattern and (**b**) absorption spectra of (PEA)_2_PbI_4_/GaN, optical band gaps of (**c**) (PEA)_2_PbI_4_ and (**d**) GaN.

The XRD patterns of the (PEA)_2_PbI_4_/GaN heterojunction and its individual regions were analyzed to determine their crystal structures, as shown in Figure 2a. The results reveal diffraction peaks corresponding to the (004), (006), (008), (0010), and (0012) planes of (PEA)_2_PbI_4_ (indicated by the red curve) [23,24], as well as the (0002) plane of GaN (indicated by the yellow curve) [19,25], consistent with previous studies. In the blue curve of the heterojunction, the diffraction peaks of both (PEA)_2_PbI_4_ and GaN can be observed, with a slight decrease in peak intensity due to the formation of the heterointerface. This indicates that the (PEA)_2_PbI_4_ film fabricated via spin coating can uniformly cover the GaN surface, with the interaction between the semiconductor materials primarily governed by weak interfacial forces, thus forming a stable heterojunction structure.

The absorption spectra, shown in Figure 2b, demonstrate that the (PEA)_2_PbI_4_/GaN heterojunction effectively combines the light absorption contributions of both (PEA)_2_PbI_4_ and GaN, indicating the formation of a good interfacial contact. In Figure 2c,d, the optical band gaps of the direct bandgap materials (PEA)_2_PbI_4_ and GaN, determined from the absorption spectra using Tauc’s principle [26], are 3.36 eV and 2.35 eV, respectively. The Tauc equation is given by (αhν)^2^ = C·(hν − Eg), where α is the absorption coefficient, hν is the incident photon energy, and C is a constant [27].

**Figure 3:**
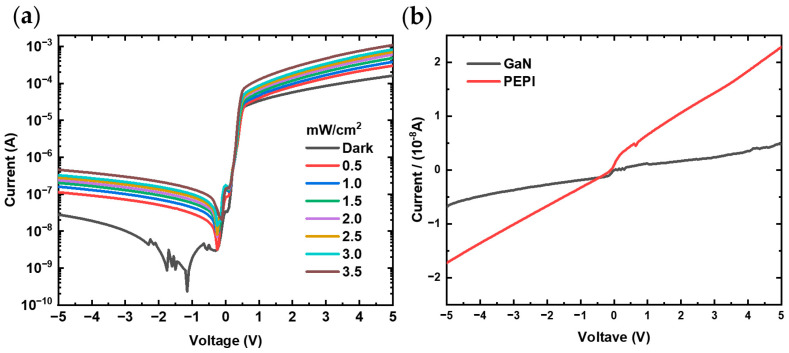
(**a**) I–V curves of (PEA)_2_PbI_4_/GaN heterojunction ultraviolet photodiodes under different light intensities, (**b**) I–V curves of (PEA)_2_PbI_4_ and GaN ultraviolet in the dark.

Based on the prepared high-quality heterojunction, the current–voltage characteristics of the heterojunction are first studied. Figure 3a illustrates the typical current–voltage (I–V) characteristics of the (PEA)_2_PbI_4_/GaN heterojunction. Under dark conditions, it demonstrates a clear rectifying effect, with a rectification ratio of 5.8 × 10^3^ at ±5 V. However, the I–V characteristics of (PEA)_2_PbI_4_ and GaN individually, as shown in Figure 3b, reveal that both materials exhibit almost no rectifying effect and demonstrate purely resistive behavior with nearly ohmic contacts under both positive and negative voltages in the dark. This indicates that the device does not exhibit diode characteristics when these materials are used independently. When the two indium electrodes are placed on (PEA)_2_PbI_4_ and GaN, respectively, it indicates that the rectifying behavior originates from the interaction between the two materials at the heterojunction interface. This observation confirms that a heterojunction diode has been successfully fabricated.

This heterojunction photodiode demonstrates a low dark current, which is a key factor in reducing noise. A lower dark current translates to reduced noise levels, an enhanced signal-to-noise ratio, and improved sensitivity. Furthermore, the low dark current contributes to the improved sensitivity of the photodiode.

The photoresponse of the heterojunction was further evaluated by analyzing its I–V characteristics under illumination from a 365 nm LED light source, based on the absorption spectrum, to assess its UV detection performance. The light intensity was systematically varied across 0.5, 1.0, 1.5, 2.0, 2.5, 3.0, and 3.5 mW/cm^2^.

The heterojunction maintains a strong rectifying effect, under illuminated conditions of 365 nm. Compared to the dark current, the photocurrent at 5 V increases significantly, reaching the mA range. Notably, the photocurrent under negative bias shows even better performance as shown in Figure 3a. This demonstrates that the heterojunction ultraviolet photodetector can operate in dual modes: a depletion mode under reverse bias and a photoconductive mode under forward bias. The ordinary p-n junction diode detector generally operates under reverse bias. However, when the diode is not fully saturated under forward bias or when the semiconductor material exhibits a strong response to the irradiation source, the diode can respond under both forward and reverse bias. This phenomenon is more common in wide-bandgap semiconductor devices, which inherently have low dark current [22].

Under forward bias, the depletion layer becomes thinner, and in photoconductive mode, the detector behaves similarly to a photoconductive resistor, where the transport mechanism can be explained by photoconductive theory. Conversely, under reverse bias, the depletion layer becomes thicker, and the transport mechanism can be explained by thermionic emission theory [28]. The corresponding formulas are expressed as the follow equations:

(1) $$I=I_{0}\;\left[\exp\left(\frac{qV}{nkT}\right)-1\right]$$

(2) $$I_{0}=AA^{*}T^{2}\exp\left(-\frac{\varphi_{B}}{kT}\right)$$

where *I*_0_ is the saturation current, *A* is the effective area of the metal-semiconductor contact, A* is the effective Richardson constant, *n* is the ideality factor, *k*_B_ is the Boltzmann constant, and *φ*_*B*_ represents the barrier height at the heterojunction interface. The difference in carrier transport mechanisms under forward and reverse bias enables the detector to achieve dual-mode detection.

To evaluate the photodetection capability of the heterojunction device, this study employs the photocurrent-to-dark current ratio (*PDCR*), responsivity (*R*), and external quantum efficiency (*EQE*) as key indicators to characterize the response characteristics of the photodiode and assess the performance of the detector.

The *PDCR* is an important metric for evaluating the sensitivity of a photodetector to a specific wavelength of ultraviolet light, and it is expressed as follows [14]:

(3) $$PDCR=\frac{I_{\mathrm{photo}}-I_{\mathrm{dark}}}{I_{\mathrm{dark}}}$$

where *I*_photo_ is the photocurrent and *I*_dark_ is the dark current.

Responsivity (*R*) is used to evaluate the light response capability of the photodetector and can be expressed as follows [29,30]:

(4) $$R=\frac{I_{\mathrm{photo}}-I_{\mathrm{dark}}}{P\cdot S}$$

where *P* is the light intensity per unit area and *S* is the effective illuminated area of the photodetector, which in this study is 0.2 cm^2^.

The rectifying ratio as the ratio of the current under forward bias to the current under reverse bias, $I_{\mathrm{forward}}/I_{\mathrm{reverse}}$, where $I_{\mathrm{forward}}$ is the current measured at a specific forward bias voltage, and $I_{\mathrm{reverse}}$ is the current measured at the same magnitude of reverse bias voltage.

Specific detectivity (D*) is an important parameter in every detector, when considering the contributions of dark current as a primary source of noise, D* can be rewritten as follows:

(5) $$D*=\frac{R\sqrt{S}}{\sqrt{2eI_{\mathrm{dark}}}}$$

where *e* is the elemental charge.

The external quantum efficiency (*EQE*) is an important parameter that represents the efficiency of photon-to-electron conversion, and its formula is given by the following:

(6) $$EQE=\frac{hcR}{e\lambda }$$

where *h* is Planck’s constant (6.626 × 10^−34^ J∙S), *c* is the speed of light, and *λ* is the wavelength of the incident light, which is 365 nm in this study.

**Figure 4:**
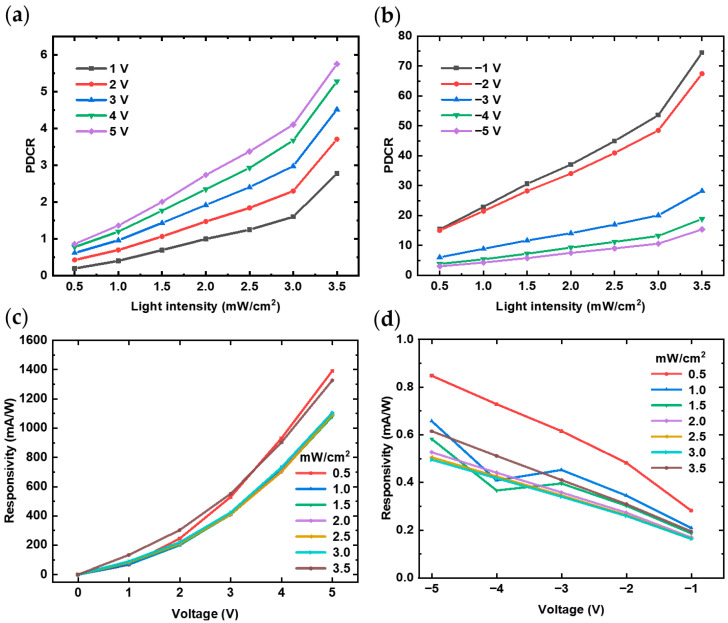
Parameters of (PEA)_2_PbI_4_/GaN heterojunction under different voltages and light intensities: (**a**) *PDCR* under positive voltages; (**b**) *PDCR* under negative voltages; (**c**) responsivity under positive voltages and (**d**) responsivity under negative voltages.

Figure 4a,b shows the photocurrent-to-dark current ratio (*PDCR*) of the photodetector under different voltages and light intensities. Under the same power density of 3.5 mW/cm^2^ of 365 nm light, the *PDCR* is 6 at +5 V and 13.2 at −5 V, demonstrating that the ultraviolet photodetector can achieve dual-mode operation. The *PDCR* value of the photodetector under forward bias is lower than that under reverse bias. This may be due to the different carrier transport mechanisms under forward and reverse bias, as well as the rectifying effect of the heterojunction.

Figure 4c,d presents the responsivity of the detector under varying voltages when exposed to 365 nm ultraviolet light. The responsivity increases with bias in both forward and reverse directions, and this trend remains consistent regardless of light intensity. At a forward bias of 5 V, a high responsivity of 1.39 A/W is achieved even under a low light intensity of 0.5 mW/cm^2^. Further calculations reveal a corresponding specific detectivity of 8.71 × 10^10^ Jones and an *EQE* of 472%. In addition to the absorption by GaN, (PEA)_2_PbI_4_ can generate multiple charge carriers (electron–hole pairs) per incident photon at 365 nm, due to the enhanced photon absorption and charge collection efficiency of the device, leading to the *EQE* exceeding 100%. It is noteworthy that although the responsivity at 0 V appears to be quite low, it can still reach approximately 0.5 mA/W, which is attributed to the high responsivity in the powered state. Additionally, a high rectification ratio of over 2.7 × 10^3^ is observed even at a low light intensity when a ±5 V bias is applied. These performance metrics clearly demonstrate the promising electrical and optical properties of the (PEA)_2_PbI_4_/GaN heterojunction UV photodetector fabricated in this study.

**Figure 5:**
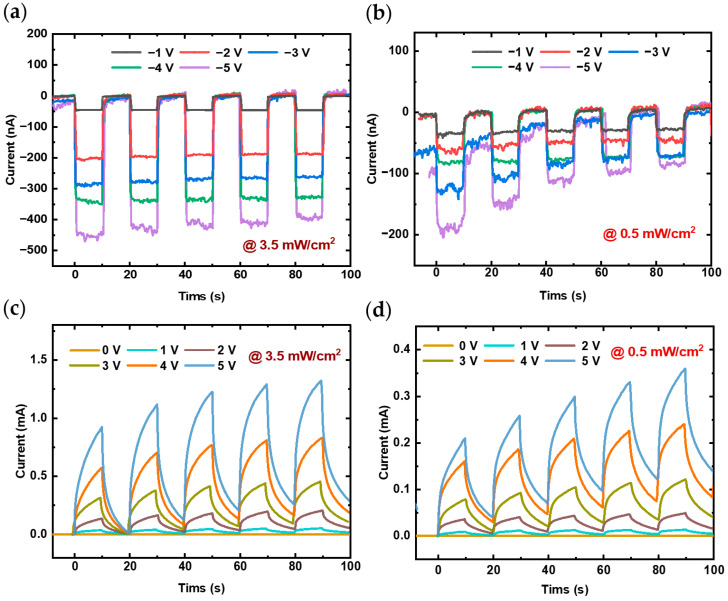
Transient photoresponse characteristics (I-t curves) of (PEA)_2_PbI_4_/GaN heterojunction under forward bias or reverse bias at the light intensities of (**a**,**c**) 3.5 mW/cm^2^, (**b**,**d**) 0.5 mW/cm^2^.

To investigate the stability and variation of photocurrent over time during ultraviolet light switching, the transient photoresponse was measured at different light intensities, as illustrated in Figure 5. These results effectively demonstrate both the stability of the photocurrent with the switching of the light source and changes in light intensity over time, highlighting the correlation between photoresponse and light intensity.

**Figure 6:**
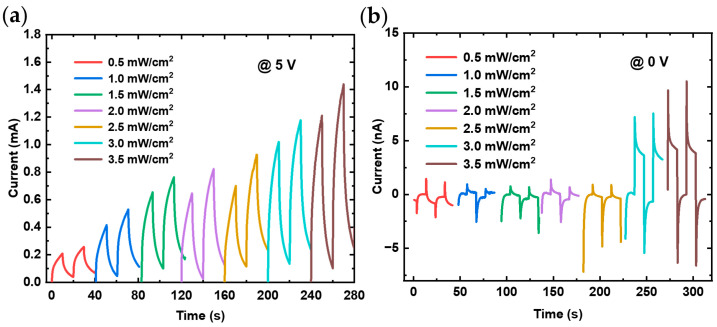
Transient photoresponse characteristics (I-t curves) of (PEA)_2_PbI_4_/GaN heterojunction under different light intensities for (**a**) at 5 V bias and (**b**) at zero bias.

In the I-t curves, the response behavior was measured over five cycles at varying voltages using alternating 10 s intervals of light exposure and darkness. Figure 5a,c presents the results under a light intensity of 3.5 mW/cm^2^, while Figure 5b,d illustrates the results at a lower intensity of 0.5 mW/cm^2^. For a given UV light intensity, increasing the voltage results in a higher photocurrent, a trend observed in both forward and reverse bias conditions as illustrated in Figure 5. Under a UV light intensity of 3.5 mW/cm^2^, increasing the voltage across the heterojunction from 1 V to 5 V increases the maximum photocurrent from 0.18 mA to 1.22 mA, as shown in Figure 5c. Similarly, under reverse bias from −1 V to −5 V, the photocurrent changes from 45 nA to 418 nA, as depicted in Figure 5a. This enhancement occurs because higher voltage boosts carrier drift velocity and facilitates the release of carriers trapped by surface defects. On the other hand, at constant voltage, increasing light intensity excites more electrons to transition into the conduction band, resulting in a higher photocurrent. Thus, the stronger UV light generates a larger photocurrent for both forward and reverse bias, as demonstrated in the comparisons between Figure 5a,b and Figure 5c,d. Under the 5 V voltage and 365 nm ultraviolet light with an irradiation power density increasing from 0.5 to 3.5 mW/cm^2^, the repeatability measure results of the photoresponse, as shown in Figure 6a, indicates a rise in the photocurrent from 0.3 mA to 1.3 mA. It can be observed that there is a certain degree of deviation in the repeatability of the photoresponse, and as the light intensity increases, the relative offset decreases. This effect may be attributed to defects on the surface of the heterojunction, which capture minority carriers, thereby limiting carrier recombination and extending the recombination time. This increases the photocurrent, resulting in a persistent photoconductive effect. Consequently, the un-recombined carriers accumulate during subsequent light exposure, which somewhat reduces the repeatability of the photoresponse. To improve experimental repeatability, the synchronous integration technique effectively mitigates the effects of charge carrier detrapping, enhancing the consistency of the photoresponse [31,32]. Nonetheless, it can still be considered that the fabricated heterojunction photodetector exhibits a certain potential for repeatability in its photoresponse.

Without an applied external voltage, the transient response of the photodetector was measured under this condition, as shown in Figure 6b. It is noteworthy that in the absence of an external voltage, the photogenerated carriers cannot be rapidly collected by the electrodes due to the internal electric field, leading to more pronounced overshoot phenomena in the photocurrent performance as the light intensity increases. This transient photocurrent is partly attributed to trap-mediated space-charge effects observed across a wide range of photoconductive devices, as highlighted in previous studies [33]. Additionally, the I-t curves exhibit alternating positive and negative responses. This could be attributed to the presence of defects on the surface during the heterojunction fabrication process. Although these defects do not alter the basic shape of the energy band diagram of the heterojunction, they do participate in carrier transport. As a result, when the light is turned off, electrons may tunnel through the barrier via interface states into the P-region, causing the dark current to be positive. Meanwhile, the photocurrent, controlled by the built-in electric field, is negative, leading to alternating positive and negative values in response to the switching of light.

**Figure 7:**
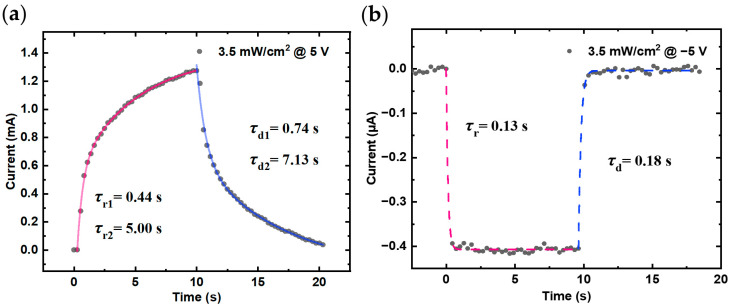
(**a**) and (**b**) are the fitted response time at light intensity of 3.5 mW/cm^2^ in ±5 V, repectively. The red lines (solid and dashed) represent the rise, while the blue lines represent the fall.

To further analyze the photoresponse, the rising and falling edges of the I-t curves under an illumination intensity of 3.5 mW/cm^2^ and a bias of ±5 V were fitted based on exponential relations, as shown in Figure 7. The rise time (*τ*_r_) and decay time (*τ*_d_) were extracted using an exponential relaxation equation to examine the photogeneration and recombination processes [34]:

(7) $$I=I_{0}+Ae^{-\frac{t}{\tau_{1}}}+Be^{-\frac{t}{\tau_{2}}}$$

where *I*_0_ is the steady-state photocurrent, *A* and *B* are the constants, *t* is time, and *τ*_1_ and *τ*_2_ are the corresponding relaxation time constant.

Based on the photocurrent response under a 5 V positive bias, the photoresponse was fitted using Equation (6), yielding rise and decay time constants of *τ*_r1_ = 0.44 s, *τ*_r2_ = 5.00 s, *τ*_d1_ = 0.74 s, and *τ*_d2_ = 7.13 s, as shown in Figure 7a. Here, subscripts 1 and 2 correspond to the coexisting processes of photoexcitation and defect trapping in the photoresponse. Under forward bias, the defect trapping process dominates, resulting in a slower photocurrent saturation due to the presence of defect traps. Under the same illumination conditions, when a bias of −5 V is applied, the photodetector exhibits a faster temporal response, as shown in Figure 7b. The instantaneous photoexcitation causes the photocurrent to rapidly increase to saturation, with band-to-band transitions dominating [14,20]. A single-exponential fit is therefore more appropriate, yielding a rise time of *τ*_r_ = 0.13 s and a decay time of *τ*_d_ = 0.18 s. Additionally, in the forward photoconductive mode, the forward bias accelerates carrier collection but also increases carrier concentration, leading to a higher recombination rate and reduced photocurrent response speed. In contrast, the thicker depletion region in reverse depletion mode decreases carrier recombination, resulting in a more stable overall response despite a lower photocurrent. Thus, the higher recombination rate in the forward mode results in a slower response speed compared to the reverse mode [35] The performance comparison between the perovskite/GaN heterojunction photodetector in this study and other devices reported in the literature is summarized in Table 1. The results indicate that our heterojunction demonstrates advantages in terms of higher responsivity and faster response speed.

**Figure 8:**
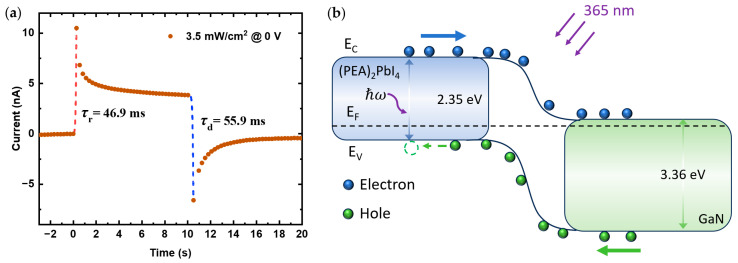
(**a**) The fitted response time at light intensity of 3.5 mW/cm^2^ and 0 V, the red and blue dots represent the rise and fall, respectively. (**b**) the band diagram of the heterojunction photodetector at zero bias.

The self-powered photocurrent response at an illumination intensity of 3.5 mW/cm^2^ is analyzed and shown in Figure 8a. As can be observed, the rise and decay time constants are 46.9 ms and 55.9 ms, respectively. These results indicate that the photodetector exhibits relatively fast temporal responses even under low light conditions. The observed rise and decay times suggest that the device can efficiently track changes in light intensity, making it suitable for applications that require rapid response times.

To further elucidate the detection mechanism of the heterojunction UV photodetector, diffusion theory can be applied as shown in Figure 8b. By referencing the conduction and valence band positions of (PEA)_2_PbI_4_ and GaN from the literature [35,38], a type-II band alignment is formed between them. When (PEA)_2_PbI_4_ and GaN come into contact, the difference in their Fermi levels causes electrons to transfer from the n-GaN side, with a higher Fermi level, to the p-(PEA)_2_PbI_4_ side, with a lower Fermi level, while holes move in the opposite direction. This process continues until thermal equilibrium is reached, forming a space charge region and generating a built-in electric field at the interface, directed from GaN towards (PEA)_2_PbI_4_. Therefore, self-powered operation can be achieved without the need for an external voltage. As the external voltage increases, the photocurrent exhibits corresponding photoresponse behaviors under both forward and reverse bias conditions. These photoresponse characteristics highlight the performance of the (PEA)_2_PbI_4_/GaN heterojunction diode photodetector fabricated in this study. The unique combination of perovskite materials and wide-bandgap semiconductors, along with the demonstrated photoresponse behaviors, makes it promising for UV light detection, environmental monitoring, and optoelectronic devices.

## 4. Conclusions

This study investigates a UV photodiode detector based on a (PEA)_2_PbI_4_/GaN p-n heterojunction. A uniform (PEA)_2_PbI_4_/GaN heterojunction was successfully fabricated using a single-step spin-coating method. The detector demonstrates effective operation under both positive and negative bias conditions, exhibiting dual-response characteristics under 365 nm UV illumination. The responsivity and detectivity under positive bias reach 1.39 A/W and 8.71 × 10^10^ Jones with rise and decay times of 0.13 s and 0.18 s. Benefiting from the type-II band alignment, the built-in electric field at the interface enables self-powered operation, with a rapid rise time of 46.9 ms and a decay time of 55.9 ms. These results underscore the rich photoelectrical response characteristics of the (PEA)_2_PbI_4_/GaN heterojunction, offering valuable insights and guidance for the future design and application of optoelectronic devices that integrate perovskite materials with wide-bandgap semiconductors.

## Figures and Tables

**Table 1:** Comparison of the performances of perovskite/GaN heterojunction photodetectors.

	Dark Current (A)	R (mA/W)	D* (Jones)	Rise Time/Decay Time (s)	Refs.
CuI/CsCu_2_I_3_/GaN	3.6 × 10^−10^	71.7	3.3 × 10^12^	8.8/0.32	[19]
CsPbCl_3_/GaN	2.42 × 10^−9^	11.5	5.82 × 10^10^	0.56/0.52	[36]
MAPbBr_3_/GaN	3.76 × 10^−7^	1.1 × 10^−4^	4.25 × 10^11^	0.21/0.44	[18,37]
BA_2_PbI_4_/GaN	0.91 × 10^−9^	——	1.08 × 10^11^	——	[38]
(PEA)_2_PbI_4_/GaN	2.34 × 10^−10^	1390	8.71 × 10^10^	0.13/0.18	This work

## Data Availability

The original contributions presented in the study are included in the article, further inquiries can be directed to the corresponding author.
